# IL8 Gene Polymorphism SNP rs4073 analysis between HTLV-1 Associated Myelopathy/Tropical Spastic Paraparesis and HTLV-1 Carriers

**DOI:** 10.1186/1742-4690-12-S1-O36

**Published:** 2015-08-28

**Authors:** Jorge Rúa, Jason Rosado, Giovanni Lopez, Carolina Alvarez, Daniel Clark, Eduardo Gotuzzo, Michael Talledo

**Affiliations:** 1 Laboratorio de Epidemiología Molecular, Universidad Peruana Cayetano Heredia, Lima, Perú; 2 Instituto de Medicina Tropical Alexander von Humboldt, Universidad Peruana Cayetano Heredia, Lima, Perú; 3 Facultad de Ciencias y Filosofía, Laboratorios de Investigación y Desarrollo (LID), Universidad Peruana Cayetano Heredia, Lima, Perú; 4 Facultad de Medicina, Universidad Peruana Cayetano Heredia, Lima, Perú

## 

Human T-cell lymphotropic Virus Type I (HTLV-1) is a retrovirus that generates an inflammatory response that can trigger a neurological disease called HTLV-1-associated myelopathy/tropical spastic paraparesis (HAM/TSP) which appears in 5-10% of infected people around the world. This inflammatory process is due the action of anti-Tax CD8+ lymphocytes that employ cytokines in order to stop the predominant infiltration of infected CD4+ T lymphocytes. IL8 is a pro-inflammatory cytokine that induces the activation and migration of neutrophils towards the sites of infection and it is continuously expressed in HTLV-1 infected cells. The SNP rs4073, located in the promoter of IL-8 gene, has been found associated to some inflammatory diseases and we hypothesized that it could be also a risk factor for the HAM/TSP development. The aim of our study was to evaluate the association of IL8 gene polymorphism SNP rs4073 and the presence of HAM/TSP. The study includes 243 infected subjects between asymptomatic (AC) and HAM/TSP patients. The genotyping of the polymorphisms was performed using the tetra-primer ARMS-PCR (Amplification Refractory Mutation System) method, 37 ancestry informative markers (AIMs) were used to correct by ethnicity and to avoid false positive results due to population stratification effect. The statistical analysis was conducted using the chi-square and Kruskal-Wallis test for possible differences among the groups. Linear and logistic regression analysis was performed using age, gender and ethnicity. The results show that none of the SNP rs4073 genotypes achieved a significant difference between AC and HAM/TSP, neither significant difference was found with proviral load (p>0.05). Our results suggest that the increase of the inflammatory response in HAM/TSP patients may not be caused by the SNP rs4073 in the promoter site of IL8, but the over expression of these gen could be possibly manage by another SNP or any other mutation in the enhancer region.

